# Exosome-Mediated Therapeutic Strategies for Management of Solid and Hematological Malignancies

**DOI:** 10.3390/cells11071128

**Published:** 2022-03-27

**Authors:** Alessandro Allegra, Claudia Petrarca, Mario Di Gioacchino, Marco Casciaro, Caterina Musolino, Sebastiano Gangemi

**Affiliations:** 1 Division of Hematology, Department of Human Pathology in Adulthood and Childhood “Gaetano Barresi”, University of Messina, 98125 Messina, Italy; cmusolino@unime.it; 2 Center for Advanced Studies and Technology, G. D’Annunzio University, 66100 Chieti, Italy; claudia.petrarca@unich.it; 3 Department of Medicine and Aging Sciences, G. D’Annunzio University, 66100 Chieti, Italy; 4 Institute for Clinical Immunotherapy and Advanced Biological Treatments, 65100 Pescara, Italy; 5 Unit of Allergy and Clinical Immunology, Department of Clinical and Experimental Medicine, School of Allergy and Clinical Immunology, University of Messina, 98125 Messina, Italy; marco.casciaro@unime.it (M.C.); gangemis@unime.it (S.G.)

**Keywords:** extracellular vesicles, exosome, cancer, hematological malignancy, cell communication, chemoresistance, microRNA, drug delivery, immune response

## Abstract

Exosomes are small membrane vesicles of endocytic origin containing cytokines, RNAs, growth factors, proteins, lipids, and metabolites. They have been identified as fundamental intercellular communication controllers in several diseases and an enormous volume of data confirmed that exosomes could either sustain or inhibit tumor onset and diffusion in diverse solid and hematological malignancies by paracrine signaling. Thus, exosomes might constitute a promising cell-free tumor treatment alternative. This review focuses on the effects of exosomes in the treatment of tumors, by discussing the most recent and promising data from in vitro and experimental in vivo studies and the few existing clinical trials. Exosomes are extremely promising as transporters of drugs, antagomir, genes, and other therapeutic substances that can be integrated into their core via different procedures. Moreover, exosomes can augment or inhibit non-coding RNAs, change the metabolism of cancer cells, and modify the function of immunologic effectors thus modifying the tumor microenvironment transforming it from pro-tumor to antitumor milieu. Here, we report the development of currently realized exosome modifiers that offer indications for the forthcoming elaboration of other more effective methods capable of enhancing the activity of the exosomes.

## 1. Introduction

Biological cell-to-cell interactions take place to preserve the homeostasis of the organisms. Extracellular vesicle (EV)-intermediated cell communication has particular importance in this field as these elements’ capacity to carry and deliver a diversity of substances from the generating cell to recipient cells [1].

Exosomes are nanometer-sized EVs generated by the internal budding of the membrane of endosomal multivesicular bodies. Exosomes were previously considered ‘trash bags’ for cellular elements to reject discarded proteins. Instead, in the last few years, they have appeared as a fundamental method for controlling a large variety of cellular activities, such as growth, programmed cell death, and angiogenetic dynamics. Moreover, exosomes have been reported to play a central role in regulating innate and adaptive immune responses, contributing to antigen presentation in connection with major histocompatibility complex (MHC) molecules [2,3].

Several experimental findings recently confirmed exosome participation in different diseases such as autoimmune pathologies, neurodegenerative diseases such as Alzheimer’s and Parkinson’s, infective diseases such as tuberculosis and HIV, and cardiovascular disorders [4].

Exosomes are involved in cancer development and diffusion and could be essential in provoking the phenomenon of chemoresistance. Evidence suggested that cell-derived EVs could mimic their parental cells, possessing the same pro-tumor and anti-tumor effects, and inherent tumor tropism. Therefore, cell-derived EVs can be a cell-free cancer treatment alternative [5].

For this motive, numerous efforts have been performed to intervene on neoplasms modifying exosome functions, exploiting exosomes that have a cell-specific tropism, determined by the elements present on their external face, directing them to precise cells or tissues [6]. It has also been shown that the immune systems do not impede endogenous exosome trafficking as they can evade immune identification and removal, unlike exogenous vesicles. Furthermore, exosomes have a protein membrane structure that facilitates effective absorption towards the cells to which they are directed, modifying their mimetic structures or arming them with different drugs or siRNAs [7]. A further therapeutic approach for tumors can be attempted by modifying pro-tumorigenic instructions contained in the exosomes or changing their load and function to prevent the onset or spread of the tumor [8,9,10].

The use of exosomes and their modulation for the treatment of solid neoplasms and hematological tumors is the subject of this review, which examines both in vitro and in vivo experiments and the still few clinical studies that have employed exosomes in the treatment of oncological diseases.

## 2. Exosomes and Breast Cancer

Breast cancer (BC) is the most frequent form of tumor detected in the female sex, with 2.1 million cases every year. It is responsible for the most significant amount of cancer-correlated death in women, with an overall 15% death provoked by tumor annually [11].

It has been reported that an augmented concentration of exosomes happens in the BC cells [12,13]. Exosomes have a central role in cancer cell growth, epithelial to mesenchymal transition (EMT), tumoral angiogenesis, and cancer diffusion in BC patients [14]. EMT is a process by which epithelial cells lose their cell polarity and cell–cell adhesion, and gain migratory and invasive properties to become mesenchymal stem cells; these are multipotent stromal cells that can differentiate into a variety of cell types. EMT is essential for numerous developmental processes including the initiation of metastasis in cancer progression. Exosomes appear to play a vital role in this process. Cancer-associated fibroblasts (CAFs) secreted exosomes promoted cell invasion and chemotherapy resistance by promoting cell stemness and EMT in cancer cells. Moreover, CAFs secreted exosomal miR-92a-3p promoted cell stemness and EMT and inhibited cell apoptosis, leading to metastasis and chemotherapy resistance in cancer cells [15].

BC cell-originated exosomes enclose several different cancer-exclusive nucleic acids and proteins, which are transferred and integrated by adjacent cells, such as mesenchymal stem cells (MSC). Consequently, MSC can be re-conditioned by alteration of altering their physiological trophic capacities into pro-tumoral functions. On the other hand, absorption of MSC-derived exosomes by tumor cells can modify the function of tumor cells, comprising BC cells. Thus, MSC-derived exosomes can transmit the input to block or to promote cancer proliferation, and the reciprocal transmission of exosomes between MSC and tumor cells modifies tumor cell behavior (Table S1).

Different approaches have been implemented to perform exosome-based treatment in BC subjects. The knowledge of exosome genesis has allowed to generate exosomes from other cell lines labeled with monoclonal antibodies to target BC cells [16]. For instance, loading MSC544 exosomes with different drugs enable them to be transmitted directly to tumor cells with an enormous therapeutic impact [17], As reported above, we can load exosomes with miRNAs or miRNAs regulators, and some of these miRNAs, called anti-oncomirs, can negatively control the expression of the oncogenes so blocking tumor diffusion [18]. It follows that an exosome armed with an anti-oncomir can reduce the protumor effect of a pro-oncogenic miRNA [19]. For instance, MSCs-originated exosomes charged with locked nucleic acid (LNA) anti-miRNA 144-3p can drastically reduce the expression of oncogenic miRNA-150 and miRNA-144-3p in MCF-7 stem-like tumor cells. In this case, the reduction in oncomirs induces a decrease in tumor cells’ clonogenicity by increasing the number of target genes both in vitro and in vivo [20].

Similarly, miRNA-106a-5p is augmented in triple-negative breast cancer (TNBC) cells and is positively associated with the cancer grade, suggesting a bad outcome for TNBC patients. MSCs can transfer miRNA-106a-5p into TNBC cells via exosomes. Several experiments demonstrated that exosomal(exo)-miRNA-106a-5p produced by MSCs facilitates cancer diffusion in TNBC cells. However, administration of lncRNA HAND2-AS1 under-expressed in TNBC cells can reduce the production of exo-miRNA-106a-5p from MSCs, suppressing TNBC development [21].

In a different experiment, miRNA-126, which efficiently controls the activation of oncogenic genes and inhibits the signaling of PTEN/PI3K/AKT, has prevented the diffusion of BC [22].

In addition to MSCs, macrophages have a role in the exosome-based treatment of BC. M2 macrophages are the most numerous cell types in the BC milieu and have a relevant effect on cancer diffusion. A study demonstrated that exosomes effectively transport miRNA-130 into macrophages, followed by an increase in M1 molecules and chemokines, such as TNF-α, IL-1β, Nos2, CD86, and Irf5 and a reduction in M2 mediators such as TGF-β, IL-10, CD206, Arginase, and Ym1. Moreover, the therapy with miRNA-loaded exosomes favors the phagocytosis capacity of macrophages. Resetting macrophages from M2 to M1 has been shown to decrease the growth and diffusion of 4T1 breast cancer cells [23].

Using exosomes as transporters of molecules with therapeutic effects has been a further attempt for the therapy of BC. Biomimetic exosomes are generated by natural materials such as cell membranes from tumor cells, or other cells such as platelets, red blood cells, white blood cells, platelets, and might be used for anticancer drug delivery. These exosomes have long-lasting persistence, target-homing capacity, and biocompatibility [24]. Exosomes can be armed with anti-tumor drugs, including them under the hydrophobic core, protected from the hydrophilic extracellular matrix (ECM) milieu until exosomes discharge them into the target cell. Kalimuthu et al. reported that paclitaxel (PTX), a hydrophobic mitotic inhibitor packed inside MSC-originated exosomes, has a relevant antitumor effect in BC in vivo and in vitro [25].

Similarly, a macrophage-originated exosomes-coated poly(lactic-co-glycolic acid) nanoplatform for treating TNBC employing doxorubicin was made [26]. In this case, the surface of the exosome was charged with a peptide to target the mesenchymal-epithelial transition factor (c-Met), which is highly expressed on TNBC cells. Such modified exosome-coated nanoparticles significantly increased doxorubicin’s cellular uptake and anticancer efficacy. These nanocarriers have shown extraordinary effectiveness, causing a marked reduction in cancer proliferation and inducing the programmed cancer cells’ death in vivo [26]. Gomari et al. confirmed that exosomal delivery of doxorubicin to HER2 + BC was very effective, with decreased cancer proliferation [27], while Melzer et al. reported that the transport of paclitaxel through MSC-originated exosomes is very effective in BC cells inducing a 90% augmentation in cell toxicity respect to the usual treatment [28]. The alteration of the environment in the tumor niche causes an increase in the immune response with a more significant number of immune cells in the niche. In a BC mouse model, the same group reported that intravenous administration of paclitaxel-armed MSC544 exosomes had greater cancer-reducing capabilities than oral dispensing of paclitaxel exosomes. When epirubicin was added to MSC544-originated exosomes, the anticancer response was found to be effective in several tumor cell cultures [29].

Furthermore, the exosomes can be labeled with specific antibodies, thus obtaining a drug-antibody conjugate which determines a greater specificity and efficacy of the drugs. Trastuzumab emtansine (T-DM1) is an antibody–drug conjugate that carries a cytotoxic drug (DM1) to HER2-positive cancer. The transport of DM1(T-DM1)-originated trastuzumab-labeled exosomes to BC cells exhibited increased toxicity against cancer cells. It has been speculated that this effect is due to a stimulation of caspase-mediated programmed cell death in BC cells [30].

Exosomes can also be charged with a series of different molecules such as carboplatin, aquaporin, and aspirin. Experiments were made with MSC-originated exosomes treated to obtain MSCCXCR4 + TRAIL (tumor necrosis factor-related apoptosis-inducing ligand) transduced with CXCR4, the most frequently observed chemokine receptor in tumor cells. It turned out that exosomes-CXCR4 + TRAIL had a synergistic effect with carboplatin in a SCID mouse model MDA-MB-231Br, setting the stage for a new approach for the therapy of BC brain metastases [31]. Promising impacts on the treatment of BC metastases were also achieved when exosomes were modified to transport aquaporin affecting miRNAs and IL-4 receptor-binding peptide [32].

Non-cytotoxic molecules can also be employed to modify exosomes. Recent findings suggest that aspirin could increase BC subjects’ survival by 20% and significantly improve therapy outcomes through a COX-1-dependent effect and rapid alteration of epidermal growth factor and EGF receptor internalization [33]. The anticancer actions of nanoamorphous exosomes derived from aspirin-loaded mammary cells have been analyzed in vitro and in vivo [34]. These exosomes were absorbed through clathrin-dependent and independent endocytosis with considerably increased cytotoxicity of aspirin to BC cells through increased programmed cell death and autophagy. Animal experiments have shown that this exosomal platform effectively transfers aspirin to BC cells in vivo. It is relevant that this exosomal system facilitates the eradication of cancer stem cells. Furthermore, the effects of these exosomes on tumor cells were amplified by coupling an aptamer specifically targeted to the epithelial cell adhesion molecule protein, a type I transmembrane glycoprotein.

The metabolic characteristics of neoplastic cells are different from those of healthy cells, which generally die in hypoxic situations. In contrast, tumor cells in a hypoxic environment survive through changes in the gene expression program [35]. Given the relevance of hypoxia in cancer cells’ survival, a study tested the anticancer actions of oxygenated water to reduce hypoxia and cancer-derived exosomes to affect cancer cells. Balb/c mice of the BC model were treated with oxygenated water administered through an intratumoral (IT) or intraperitoneal (IP) route and/or exosome (TEX) [36]. Results revealed a substantial decrease in cancer dimension, the most significant concentrations of IFN-*γ* and IL-17, and the slightest concentrations of HIF-1 alpha, MMP-2, MMp-9, IL-4, FoxP3, and VEGF the IT+IP+TEX-treated group [36].

Exosomes also participate in a paracrine reduction in cancer proliferation through inhibition of angiogenesis in BC cells. MSC-originated exosomes cause a relevant and dose-dependent decrease in vascular endothelial growth factor (VEGF) production by changing BC cells’ mTOR/HIF-1α signaling axis. Moreover, miRNA-100 transported by MSC-derived exosomes to BC cells induces a decrease in VEGF. The supposed effect of exosomal miRNA-100 transport in controlling VEGF production was confirmed by the capacity of anti-miRNA-100 to contrast the repressing action of MSC-originated exosomes on the production of VEGF in BC-derived cells [37].

Exosomes have also been used successfully in particular cancer treatment modalities such as photothermal therapy (PTT) and sonodynamic therapy (SDT). Exosomes associated with chemotherapy and PTT could represent a new strategy for treating BC. Indocyanine green (ICG) is a commonly used photosensitizer that can cause cancer cell death under 808 nm laser irradiation, with a greater antitumor effect when combined with doxorubicin. Nevertheless, the hydrophobic properties of ICG and the toxicity of doxorubicin in vivo restrain its clinical use. These problems are overcome by co-loading ICG and doxorubicin into exosomes, which further augments the efficacy of anticancer therapy. Armed exosomes, efficiently taken up by cancer cells, can increase cancer accumulation, maintaining the photothermal action of ICG and cytotoxicity of doxorubicin. ICG can induce hyperthermia under 808 nm near-infrared irradiation causing the collapse of nanovesicles with an augmented drug discharge that causes cancer cell death. In vivo experiments confirmed that armed exosomes efficiently reduce the proliferation and metastasis of BC [38].

SDT acts by generating reactive oxygen species (ROS) from sonosensitizers under ultrasound (US) exposure and is a possible substitute for PTT in tumor treatment. SDT can be applied in the therapy of deep-placed cancers which the PTT cannot achieve since the US has greater tissue penetration. The generation of carriers that can transport sonosensitizers into cancer cells without general harmfulness is essential to enable the implementation of SDT into clinical practice [39], exosomes could be a perfect choice. In a study, exosomes were employed as transporters of a sonosensitizer, ICG, the same substance reported above. The exosomes can be modified with tumor-targeting ligands. ICG-loaded exosomes (ExoICG) with folic acid (FA) as ligand significantly augmented ROS production in BC cells causing superior sonotoxicity against BC cells than free ICG, without systemic effects [40]. Remarkably, cancer proliferation in mice was considerably reduced by a single intravenous administration of the FA-ExoICG and subsequent US treatment [41].

Using exosomes armed with cytotoxic substances could be especially advantageous for the therapy of BC metastases problematic to treat with traditional chemotherapy. For instance, brain metastasis is a great challenge in the treatment of BC due to its inaccessibility. Regrettably, more than a quarter of BC subjects present brain metastasis and, unfortunately, employed anticancer drugs cannot pass through the blood–brain barrier effectively [42]. A diverse opportunity is to provoke an immune response against the BC cells or the cancer milieu. Natural killer (NK) cells are a group of lymphocytes that specifically identify and destroy tumor cells and are stimulated by the interaction between cancer cells on MHC 1 molecules. This causes the merging of NK granules to the plasma membrane, cell degranulation, and entrance of molecules into the cancer cell that consequently die [43]. NK cells also release these molecules through EVs. Cochran et al. identified granulysin, perforin, and granzymes A and B in EVs generated from NK3.3 cells, a clonal normal NK cell line. They reported that NK3.3 EVs enclose different cytolytic substances such as. K3.3-derived EVs were administered intratumorally every 3–4 days in a model of BC mice, with a decrease in tumor and caspase-mediated programmed BC cell death [44].

An interesting cancer treatment procedure was proposed by Wang et al. employing gene-delivered enzyme prodrug therapy (GDEPT) in HER2^+^ BC. EVs with specific membrane molecules able to target HER2 receptors of BC cells and enclose the prodrug 6-chloro-9-nitro-5-oxo-5H-benzo-(a)-phenoxazine and the bacterial enzyme mRNA from *E. coli* were used. Release of these substances into BC cells caused translation of the enzyme’s mRNA which operated on prodrug to generate the cytotoxic active 9-p amino-6-chloro-5H-benzo[a]phenoxazine-5-one which results in decreased BC proliferation in vitro as well as in vivo [45].

An interesting cancer treatment procedure was proposed by Wang et al. employing gene-delivered enzyme prodrug therapy (GDEPT) in HER2^+^ BC. It used EVs with specific membrane molecules able to target *HER2* receptors of BC cells and enclose the prodrug 6-chloro-9-nitro-5-oxo-5H-benzo-(a)-phenoxazine and the bacterial enzyme mRNA from *E. coli*. The release of these substances into BC cells caused translation of the enzyme’s mRNA, which operated on prodrug to generate the cytotoxic active 9-p amino-6-chloro-5H-benzo[a]phenoxazine-5-one, resulting in decreased BC proliferation in vitro as well as in vivo [45].

Studies on BC-derived exosomes aiming at cell-to-cell communication in tumor advancement, diagnosis, and therapeutic technologies may assist in further obtaining exosome-based cellular control systems for BC treatment by eliminating and/or inactivating cancer-correlated exosomes, which augment tumor progression or employing exosome-based intracellular transport of functional molecules. A critical role will be played by analyzing exosomal miRNAs expression profiles and amounts closely correlated to BC extent, type, and prognosis. Deep comprehension of such relationships and the systematization of experimental results will give a new BC identification and management approach.

### 2.1. Exosomes and Chemoresistance in Breast Cancer

Drug resistance, the main obstacle to BC therapy being correlated to a lousy outcome [46], is modulated by exosomes through control of gene expression [47,48,49]. MiRNAs could modify resistance in BC cells [50]. About 60% of patients, after early response, acquire resistance to trastuzumab, a monoclonal antibody pursuing the extracellular region of HER-2 BC [51]. In these patients, exosome-transmitted miRNA-567 reduces autophagy and augments sensitivity to trastuzumab targeting ATG5 an autophagy-correlated protein linked to BC carcinogenesis [52].

Furthermore, macrophage-originated exosomes armed with PTX, and doxorubicin can be employed to overwhelm the multidrug resistance in BC [53]. The sensitivity to doxorubicin is increased by exosome-transported miRNA-770 by decreasing the stathmin1 (STMN1) gene in vitro and in vivo [54]. It operates by regulating programmed cell death and modifying the cancer milieu. Restoration of STMN1 could partially nullify the effect of miRNA-770 [55] (Table S2).

### 2.2. Exosomes and Lung Cancer

Lung cancer (LC) is the leading cause of tumor-related death worldwide, and non-small-cell lung cancer (NSCLC) stands for about 85% of all cases of LC [56].

As in the case of BC, numerous experiments on the therapeutical use of exosomes have been performed for the LC. For instance, the inhibitory effect of miRNAs transported by exosomes was evaluated. Human cell-originated exosomes containing miRNA-497 efficiently inhibited cell proliferation and the production of genes such as VEGF, cyclin E1, yes-associated protein 1, and hepatoma-derived growth factor in A549 cells of NSCLC [57]. Remarkably, the effects of exo-miRNA-497 on angiogenetic dynamics were studied. A549 cells were co-cultured with human umbilical vein endothelial cells (HUVECs) to simulate the in vivo-like cancer milieu of NSCLC. The generation of endothelial cells and migration of cancer cells was reduced significantly compared to the control when miRNA-497-loaded exosomes were added.

A similar effect with an antineoplastic action can target lymphatic vessels, which are essential constituents of both cancer and lung microenvironments. Once cancer has taken roots, nearby lymphatic endothelial cells (LECs) are stimulated and generate novel vessels under the effect of specific signals sent by cancer cells or the milieu. In a study, Yang et al. demonstrated that glypican-5 (GPC5), a component of heparan sulfate proteoglycans, augments the expression of CTDSP1 (miR-26b host gene) through the AhR-ARNT system, and such an increase causes a reduction in lung tumor cell proliferation. Furthermore, exosomes originating from GPC5-overexpressing lung cancer cells (GPC5-OE-originated exosomes) had an inhibitory action on LECs, reducing their generation and diffusion. The mechanism of action of miRNA-26b in LEC is related to the inhibition of the protein tyrosine kinase 2 (PTK2) 3′-UTR [58].

Similarly, let-7a-5p delivered from macrophages to lung tumor cells inhibits lung cancer cell proliferation acting, via PI3Kg signaling system, on Bcl-2-like protein-1 (BCL2L1), a potent inhibitor of apoptosis. This finding was demonstrated in vitro on A549 lung cancer cells, and it has been observed that altered production of let-7a-5p modifies the survival of lung cancer patients. Moreover, altered expression of BCL2L1 considerably changes the generation of lung tumor markers such as vimentin, MYC, and EGFR [59].

Xue et al. demonstrated that the combination of miRNA-34a and K-ras siRNA enclosed in lipid polymers positively affects a lung adenocarcinoma model [60].

Moreover, the exosomes can be charged with drugs used to treat lung cancer. Antitumor effectiveness of airway-delivered exosomes armed with paclitaxel was reported in a model of murine Lewis lung carcinoma pulmonary metastases [53]. Moreover, the same authors implemented a novel exosome-based drug transport platform able to transfer drugs. Their experimentation targeted moiety aminoethylanisamide-polyethylene glycol (AA-PEG) into the exosome-PTX preparation. AA-PEG aimed at the sigma receptors, augmented in lung tumor tissues, and increased the therapeutic response [61].

A further strategy of cancer treatment can be performed by promoting the anticancer immune response. Cortez et al. reported that exosomal transfer of miRNA-34a mimics (MRX34) into a lung adenocarcinoma experimental animal model increased the number of cancer-infiltrating CD8^+^ cells and reduced the amounts of T-regulatory cells, macrophages, and tumor-infiltrating PD1^+^ T-cells [62].

Always in this area, dendritic cells (DC) vaccine-based immune treatment has been applied as a therapeutic approach for tumor therapy. However, the anticancer action of DC vaccines performed on tumor cell lysates (TCLs) has given unsatisfactory results due to cancer antigens’ modest immunogenicity. Tumor-associated exosomes (TAEs) have been studied as an encouraging opportunity for DC vaccines. In a study, authors isolated TAEs from the supernatant of cancer cell culture medium and compared the action of TAEs with TCLs on DCs. The results proved that TAEs were more efficient than TCLs in stimulating DC, causing a more robust tumor-specific cytotoxic T lymphocyte response.

Furthermore, TAEs decreased the presence of PD-L1 of DCs, thus provoking a decrease in T regulator cells in vitro. Finally, DC-TAE significantly reduced cancer proliferation and augmented survival in vivo [63]. Based on these data, a phase I clinical trial with tumor-associated antigens-loaded DC-derived exosomes in NSCLC subjects [64], and a phase II clinical trial with DC-originated exosomes for maintenance immune-treatment after chemotherapy in NSCLC were executed [65] (NCT01159288). A relevant novelty in this trial is utilizing exosomes originating from Toll-like receptor 4- or IFN-*γ*-maturated DCs. It was reported that such exosomes cause stronger T cell stimulation versus DC-originated exosomes from immature DCs. Thirty-two percent of patients showed stability of their tumor for over 4 months, while only one patient presented grade-3 hepatotoxicity [65].

Then et al. performed a study to evaluate the capacity of cryopreserved umbilical cord blood mononuclear cell-derived DCs (cryo CBMDCs) and their exosomes to modify allogeneic T cell growth and to study toxicity against A549 lung tumor cells [66]. They proved that cell lysate-pulsed DCs and their exosomes could stimulate allogeneic T cell growth and have a cytotoxic effect against A549 cells. These findings also evidenced that cryo umbilical cord blood mononuclear cells source is efficient for producing exosomes and might be useful for vaccinating against tumors.

Moreover, unconventional substances with anticancer potential can be inserted into exosomes. Employing bovine milk as a fount of exosomes, Munagala et al. [67] showed that withaferin A, a steroidal lactone, has increased anti-tumor actions in lung tumor cell lines and in in vivo lung cancer xenografts when transported through exosomes concerning free withaferin A. Similarly, exosome-armed celastrol (a plant-originated triterpenoid inhibitor of heat shock protein 90 and NF-κB) showed a more significant anticancer action in lung tumor cell xenograft experimental model for free drug [68].

Curcumin is another substance studied in anticancer treatment as it induces DNA hypomethylation, which epigenetically regulates genome stability [69]. Curcumin reduces the activity of a methyltransferase, DNMT1, so modifying the methylation of several tumor-correlated genes [70] and causes an augment of the transcription factor 21 (TCF21). In a study, exosomes derived from curcumin-pretreated H1299 cells caused an increase in TCF21 amount in BEAS-2B cells with a decrease in cancer cells proliferation [71]. Analysis performed by the GEO database showed a positive correlation between TCF21 concentrations and lung tumor subject survival.

Lung tumor-derived exosomes transferring molecular information from LC cells to cells close to them or at distant sites may activate the tumor microenvironment and accelerate tumor diffusion. Exosomes seem to intervene in the regulation of numerous processes that affect neoplastic lung cells, such as angiogenesis, apoptosis, and the immune response. Furthermore, exosomal miRNAs might be believed as noninvasive markers for precocious diagnosis of LC. Finally, several promising modalities in the treatment of LC with loading microRNAs, drugs, protein, and siRNAs inside specific antigen-targeted exosomes have been proposed.

#### Exosomes and Chemoresistance in Lung Cancer

Various exosome-associated substances can reduce resistance to drugs used in lung cancer, such as cisplatin and erlotinib. For instance, by operating as tumor suppressors, exosome-associated miRNA373, miRNA146a-5p, and miRNA512 are strongly correlated with reduced chemoresistance to cisplatin [72,73]. CAFs have been reported to be crucial stromal constituents in the cancer milieu, controlling tumor proliferation, diffusion, and chemo-response via various pathways [74]. NSCLC-originated CAFs were resistant to cisplatin administration. In a study, CAFs-originated exosomes were taken up by NSCLC cells, and exosomal miRNA-130a was transported from CAFs to NSCLC cells, causing chemoresistance. Contrariwise, knockdown of miRNA-130a inverted the effect of CAFs-originated exosomes. Moreover, it was demonstrated that pumilio homolog 2 (PUM2), an RNA-binding protein, favors the storage of miRNA-130a into exosomes. The augment or knockdown of PUM2 stimulated or decreased cancer proliferation in the xenograft mice experimental model [75]. These findings show that CAFs-originated exosomes induce cisplatin chemoresistance of NSCLC cells via transporting miRNA-130a and that PUM2 is an essential element for modifying cisplatin chemoresistance in NSCLC.

Moreover, phosphoribosyl pyrophosphate synthetase 2 (*PRPS2*) is reported as an oncogene in different tumors. *PRPS2* increases cisplatin resistance of NSCLC cells enabling exosome-mediated macrophage M2 polarization in NSCLC tissues [76].

Finally, exosomal lncRNA H19 favors erlotinib resistance in NSCLC cells [77]. In fact, H19 concentrations were augmented in erlotinib-resistant cells, while knockdown of H19 reduced cell growth. Extracellular H19 can be stored into exosomes, and Exo-H19 provoked erlotinib resistance of sensitive cells, while knockdown of H19 inverted this action. Moreover, it was demonstrated that miRNA-615-3p was a target of H19 and can link to autophagy-related gene (ATG)7. Exosomal H19 modified erlotinib resistance of NSCLC cells through targeting miRNA-615-3p to control ATG7 expression. In an experimental model, HCC827 cells transfected with sh-NC or sh-H19 lentiviral vector were injected into nude mice administered with erlotinib, demonstrating that knockdown of H19 reduced erlotinib resistance through controlling miR-615-3p and ATG7 generation in vivo.

### 2.3. Exosomes and Colon Cancer

Colon cancer (CC) is among the more frequent tumors affecting about one million people every year. Its mortality percentage ranks fourth among cancer-related deaths [78].

Managing miRNAs originating from exosomes is a possible therapeutic approach to CC. The aberrant increase in macrophages in CC tissues participates in its diffusion, and exosomal miRNA-183-5p has been reported as an oncogene. Zhang et al. studied the influence of exosomal miRNA-183-5p enhanced by M2-polarized tumor-associated macrophages (TAM) on CC cells [79]. M2-TAM-originated exosomes remarkably augmented CC cells growth and inhibited programmed cell death. It decreased miRNA-183-5p rescued M2-TAM-correlated cancer-supportive influences. Exo-miRNA-183-5p affected thioesterase superfamily member 4 (THEM4) and reduced protein production. Contrariwise, THEM4-increase consolidated miRNA-183-5p-correlated pro-tumorigenic actions and rendered inactive NF-kB and Akt systems in CC cells.

Moreover, it is interesting that cancer cells and normal cells possess different miRNAs originating from exosomes, with different consequences. A study evaluated miRNAs in exosomes derived from human CCC SW620 cells and human normal colon epithelial cells NCM460 [80]. Two groups of miRNAs were described: “high in exosome and high in cell” (HEHC) and “high in exosome but low in cell” (HELC). MiRNA-2277-3p and miRNA-26b-3p, which are representative of the different groups, have opposite effects: MiRNA-2277-3p stimulates growth and diffusion of SW620 cells by aiming Nupr1-like isoform (NUPR1L), while miRNA-26b-3p has a blocking action by targeting prefoldin 1 (PFDN1). Exosomes rich in miRNA-2277-3p have a tumor-promoting action, whereas exosomes rich in miRNA-26b-3p have no activities in tumor cells.

Other molecules can modify the activity of the non-coding genetic material, varying their pro or antineoplastic activity. For instance, Sevoflurane, an inhaled anesthetic frequently employed in clinical practice, has a remarkable effect on CC cell proliferation [81]. Circular RNA 3-hydroxy-3-methylglutaryl- CoA synthase 1 (circ-HMGCS1) is reduced in CC cells by sevoflurane and circ-HMGCS1 augment could rescue the action of sevoflurane on CC cell growth. MiRNA-34a-5p is the target of circ-HMGCS1, and miRNA-34a-5p reduction inverts the act of circ-HMGCS1 silencing on CC cell proliferation [82]. Moreover, circ-HMGCS1 knockdown reduces sphingosine-1-phosphate phosphatase 1 production through sponging miRNA-34a-5p and decreases cancer proliferation in vivo. Thus, sevoflurane blocks CC diffusion by regulating the exosome-transmitted circ-HMGCS1/miRNA-34a-5p/SGPP1 axis.

#### Exosomes and Chemoresistance in Colon Cancer

Treatment founded on 5-fluorouracil (5-Fu) is the conventional line for the therapy of CC, but chemoresistance to 5-Fu is the main reason for tumor advancement in CC subjects.

Exosomes from 5-Fu-resistant CC cells seem to stimulate angiogenesis, and exosomal growth/differentiation factor 15 (GDF15) is a potent activator of the angiogenetic dynamics by blocking the Smad pathway. Transforming growth factor (TGF)-*β*1, an inducer of the Smad pathway, could partially reduce this effect. Thus, the Exo-GDF15-Smad axis might be a new target to increase sensitivity to 5-FU in colon cancer [83].

### 2.4. Exosomes and Hepatocellular Carcinoma

Hepatocellular carcinoma (HCC) constitutes about 75% to 85% of primary liver tumors. Current data report that approximately 840,000 people experience HCC every year moreover, and about 780,000 people die from this form of tumor every year [78].

Several types of exosomes seem to have a tumorigenic effect on HCC. For instance, Sphingosine kinase 2 (Sphk2) is a relevant controller regulator in exosome genesis. Employing Sphk2 siRNA-loaded nanoparticles can decrease miRNA-21 presence into exosomes, participating in reducing the HCC pro-tumoral activity of exosomes [84].

Other data propose that MSC-originated exosomes could block HCC development, which was correlated with transporting cancer-suppressed ncRNAs [85,86]. Contrariwise, some reports stated that MSC-derived exosomes might stimulate cancer growth [87].

Lou et al. described that miRNA-122-transfected adipose mesenchymal stem cells (AMSCs) could store miRNA-122 into exosomes, and miRNA-122-loaded exosomes make HCC cells more susceptible to chemotherapeutic drugs by reducing metalloprotease domain 10, insulin-like growth factor 1 receptor, and cyclin G1 [88]. Liang et al. employed electroporation to charge miRNA-26a to HEK-293T cell-derived exosomes. These exosomes could reduce HCC proliferation by repressing proliferative factors such as cyclin-dependent kinase 6, cyclin D2, and cyclin E2 [89]. Similarly, HSC-derived exosomes armed with miRNA-335-5p inhibited HCC cell growth by targeting different epigenetic regulators, factors such as thrombospondin-1, casein kinase 1 gamma 2, CDC42, CDK2, eukaryotic translation initiation factor 2C2, eukaryotic translation initiation factor 5, LIMaK1, NRG1, polo-like kinase 2, TCF3, YBX1, and zinc finger MYND-type containing 8 [90].

Moreover, it is possible to employ siRNA-armed exosomes to treat HCC. For example, glucose-regulated protein 78 (*GRP78*), a well-known molecular chaperone in mammalian cells, is correlated with sorafenib chemoresistance but employing exosomes containing siRNA against siRNA GRP78 could inhibit sorafenib resistance in HCC [54].

Finally, exosomes originating from BM-MSCs provoke programmed cell death in HepG2 cells, while the intra-tumor dispensation of exosomes administration of exosomes in established tumors generated by subcutaneous injection of HepG2 cells in SCID mice significantly reduced tumor growth [84].

Other substances could be used to arm exosomes against HCC cells. Sinomenine (SIN), an alkaloid found in the root of the plant *Sinomenium acutum*, has an anticancer effect in vitro. A study evaluated the efficacy of combined exosomes-SIN for HCC treatment in an experimental animal model showing a more significant reduction in HepG2 cells concerning free SIN. Exo-SIN causes programmed cell death and cell cycle arrest by reducing survivin, an essential protein for the survival of cells [91].

miRNA-142 and miRNA-223 can also be transported from macrophages to liver cells by exosomes to reduce the proliferation of HCC cells [92]. Exosomal miRNA-122 produced by hepatocellular carcinoma can inhibit EMT, increase drug sensitivity, and inhibit angiogenesis by targeting LMNB2 [93,94]. Finally, Wang et al. demonstrated that LX-2 exosomes, supplemented with miR-335-5p, decrease HCC proliferation after intra-tumoral administration in MHCC97H cell xenografts [95].

#### 2.4.1. Exosomes and Immunotherapy for HCC

Recent experiments show that AD-MSC-originated exosomes can stimulate anti-HCC NK T cell responses in rat N1S1 cells (an orthotopic HCC model) [96]. This and other data suggest the possibility of HCC immunotherapy. A study demonstrated that bone marrow mesenchymal stem cells acquired relevant anticancer ability after exposure to HCC cell-originated exosomes and blocked the growth of HCC cells. 

Alpha-fetoprotein (AFP)—expressing DC-originated exosomes (DEXs) produced a powerful antigen-specific immune response and determined a decreased HCC proliferation and augmented survival of animals with carcinogen-caused HCC tumors that exhibited antigenic heterogeneousness [97]. Moreover, this type of approach was able to modify immune effectors in the cancer milieu. DEXAFP-treated HCC mice presented significantly more gamma interferon-expressing CD8^+^ T lymphocytes and augmented the concentrations of IFN-gamma and interleukin-2, with reduced CD25^+^/Foxp3^+^ regulatory T cells and reduced concentrations of interleukin-10 and transforming growth factor-beta in the cancer microenvironment. The absence of effectiveness in CD8^+^ T cell-depleted mice suggests that T cells participate in DEXAFP-mediated anticancer activity [97]. However, although it has been demonstrated that mast cells-originated exosomes increase HCC cell proliferation, HCV-E2-stimulated MC-originated exosomes block the migration of HCC cells and reduce HCC cell metastasis by repressing the ERK1/2 signaling pathway [90].

#### 2.4.2. Exosomes and Chemoresistance in HCC

Chemoresistance sometimes makes ineffective the treatment with Sorafenib of HCC patients with advanced disease [98]. It has been demonstrated that resistant HCC cell-generated exosomal miRNA-210 may be transported into non-resistant HCC cells, provoking multidrug resistance via the reduction in phosphatase and tensin homolog deleted on chromosome ten and the augmentation of the PI3K/Akt pathway [99].

miRNA-744 was reduced in exosomes originating from resistant HCC cells, and these exosomes could be absorbed by non-resistant HCC cells. The reduced concentration of miRNA-744 causes an increase in paired box 2 that favors HCC cell growth and reduces the chemosensitivity of non-resistant HCC cells [100].

TGFβ has also been associated with chemoresistance as it decreases the sensitivity of HCC cells to sorafenib or doxorubicin and modifies the delivery of both extracellular vesicles and specific long non-coding RNA (lncRNA) within these vesicles. LincRNA-ROR (linc-ROR), a stress-responsive lncRNA, was highly increased in HCC cells. Incubation with HCC-originated EVs augments linc-ROR expression and decreases chemotherapy-caused cell death via the repression of p53 [101]. These results demonstrate that exosomal ncRNAs might boost chemosensitivity in HCC.

Lou et al. evaluated if MSC-originated exosomes could operate as transporters of miRNA-199a-3p to augment HCC chemosensitivity. In this study, miRNA-199a lentivirus infection and puromycin selection generated miRNA-199a-modified AMSCs (AMSC-199a). AMSC-Exo-199a appreciably reduced chemoresistance of HCC cells to doxorubicin in vitro by altering the mTOR signaling pathway. Moreover, its effect was significantly augmented by i.v. administration in vivo [102].

HCC cells treated with exosomes and cisplatin display augmented sensitivity to cytotoxic T lymphocytes (CTLs), with prolonged survival of animals. Likely, after immunization with exosomes, FasL increases spleen lymphocytes’ binding to the Fas on target cells, inducing HCC cells apoptosis. This data suggest that exosomes can synergize with chemotherapy and remarkably augment their antitumoral effects.

On the other hand, as reported above, exosomes can also augment chemoresistance in HCC. Exosomes from two aggressive HCC cell lines were able to provoke sorafenib resistance in vitro by stimulating the hepatocyte growth factor/c-Met/Akt pathway and reducing sorafenib-caused programmed cell death [103].

Finally, Rab27B expression in 5Fu-resistant Bel7402 (Bel/5Fu) cells augments remarkably analogized drug-sensitive Bel7402 cells, and Bel/5Fu cells produced more exosomes under 5Fu stimulation. Moreover, exosomes produced by Bel/5Fu cells suggestively decrease after knocking down Rab27B, and the cellular levels of 5Fu augment, increasing its therapeutic action. The dispensation of drug efflux pump inhibitors and knockdown of Rab27B further extend the positive actions of 5Fu [104].

### 2.5. Exosomes and Melanoma

Malignant melanoma is the most severe skin cancer, and its frequency is continually increasing, and with metastatic diffusion, subjects have a negative outcome [105].

Inserting active molecules into exosomes could be a valid approach to treat melanoma. Some experiments demonstrated that MSC-originated exosomes could reduce melanoma diffusion via increased apoptosis or a cell cycle arrest with an augmentation of caspase3 cleavage and a decrease in Akt phosphorylation. Other experiments suggest that exosomes interfere with angiogenetic processes reprogramming melanoma cells and reducing VEGF expression.

In a well-designed study, mesenchymal stem cells presenting TRAIL/green fluorescent proteins (TRAIL is an essential mediator of the programmed cell death [106]) were generated, and exosomes including TRAIL protein (Exo-TRAIL) were separated. The anticancer efficacy of both MSC-originated exosomes and Exo-TRAIL was evaluated, demonstrating that the co-administration of both Exo-TRAIL and tumor cells deferred melanoma development. Moreover, the melanoma weight was significantly reduced in melanoma-bearing animals treated by multiple administrations of Exo-TRAIL while a single administration did not display a relevant anticancer effect [107].

In a different study, TRAIL-modified exosomes charging triptolide (TPL), the principal active and toxic component of *Tripterygium wilfordii Hook F*, were evaluated for the treatment of melanoma [108]. TRAIL/TPL exosomes seem able to augment melanoma targetability, decrease growth and diffusion of melanoma cells, and stimulate programmed cell death of A375 cells via triggering the extrinsic TRAIL system and the intrinsic mitochondrial system in vitro. Moreover, in vivo, intravenous administration of Exo-TRAIL/TPL remarkably reduced melanoma diffusion in a nude mouse experimental model [109].

Numerous studies have used various engineered liposomes to treat melanoma [110,111]. Similar or even better results have been achieved employing exosome-based therapy. Zhu et al. proposed a possible immunotherapeutic approach for melanoma using exosomes originated from NK with promising results [112].

However, the most innovative therapeutic attempt involves the activation of the Stimulator of Interferon Genes (STING) system associated with the innate immune response. Stimulation of the cytosolic DNA sensor STING in antigen-presenting cells (APC) causes a response to type I interferon. However, STING agonists (STINGa) have negative pharmacological characteristics, such as rapid clearance and inadequate transfer to the cytosol. McAndrews et al. used exosomes loaded with STING’s GMP-AMP cyclic agonist (iExoSTINGa) to specifically target the STING pathway in APCs with greater efficacy than STINGa alone in inhibiting B16F10 cancer proliferation. Furthermore, iExoSTINGa showed greater uptake of STINGa in dendritic cells than STINGa alone, with a significant increase in stimulated CD8^+^ T cells and an improved antitumor immune response [113].

Finally, tumor vaccination has been limited by the complex recognition of specific cancer antigens and their transport to APC. The exosome-founded cancer antigens-adjuvant co-transport technique employing genetically modified cancer cell-originated exosomes, including cancer antigens and immunostimulatory CpG DNA, was used in a murine melanoma B16BL6 [114]. Murine melanoma B16BL6 cells were transfected with a plasmid vector encoding a fusion streptavidin (SAV)-lactadherin (LA; an exosome-tropic protein) protein, yielding genetically engineered SAV-LA-expressing exosomes (SAV-exo). CpG-SAV-exo displayed active transport of exosomes with CpG DNA to murine dendritic DC2.4 cells, with stimulation of DC2.4 cells and augmented cancer antigen presentation. Immunization with CpG-SAV-exo showed greater in vivo anticancer action in B16BL6 cancer-bearing animals than the co-dispensation of CpG DNA and exosomes [114].

### 2.6. Exosomes and Ovarian Cancer

Epithelial ovarian cancer (EOC) is the leading cause of gynecological-correlated deaths and the fifth reason for tumor mortality in women [115].

Numerous studies have demonstrated the therapeutic efficacy of exosome-based therapy in ovarian cancer. For instance, macrophage-originated exosomes bearing TNF-like weak inducer of programmed cell deaths (TWEAK) reduce the motility and diffusion of EOC cells. Their action is due to the increased production of miRNA-7 with the reduction of EGFR/Akt/ERK1/2 pathway stimulation, proposing that exosomal miRNA-7 from TWEAK-activated macrophages may have encouraging therapeutic perspectives [116,117].

IRGD exosomes, generated through transfecting plasmid with cancer-targeting peptide IRGD to exosomes, were reported to interconnect with ovarian cancer cell NuTu-19 via membrane fusion specifically. IRGD exosomes armed with adriamycin (Dox) were administered to animals with ovarian tumors causing a reduction in the tumor volume [118]. Similarly, tumor-derived exosomes (TEX) loaded with staphylococcal enterotoxin B (SEB) have cytotoxic effects on ovaria tumor cell SKOV3 with remarkably reduced tumor growth and increased programmed cell death [119].

As reported above, Triptolide (TP) is a molecule that has been isolated from the herbal medicine *T. wilfordii Hook F.*, which has numerous anticancer, and immunomodulatory effects, the clinical use of which is limited due to severe side effects. It has been demonstrated that TP-enclosed exosomes stimulate programmed cell death, modify the cancer milieu by repressing M2 cancer-associated macrophages, and augment anticancer immunity. Unlike free TP, they remarkably increased the anti-tumor effects, with a reduction in side effects [120].

Interfering with miRNAs contained in exosomes has been demonstrated as a promising way to treat EOC. MiRNA-101 mimics are possible therapeutic tools for EOC therapy as it inhibits the production of brain-derived neurotrophic factor by acting on 30-UTR [121]. Different concentrations of miRNA-101 in both ovarian tissue and serum exosomes could inhibit tumor cell growth repressing genes such as zinc finger E-box-binding homeobox 2, membrane-associated ring-CH-type finger 7, suppressor of cytokine signaling 2, and enhancer of zeste 2 polycomb repressive complex 2 subunit [122].

Since omento fibroblasts are a source of exosomes [11,123], Kobayashi et al. evaluated the feasibility of using fibroblast-derived exosomes for miRNA transport [124]. MiR199a-3p-loaded exosomes (miRNA-199a-3p-Exo) significantly increased the concentration of miRNA-199a-3p in several OC cell lines, resulting in the inhibition of cell growth and spread, possibly by suppressing the expression of c-Met, a direct target of miRNA-199a-3p. Moreover, in a xenograft study, miRNA-199a-3p-Exo also remarkably reduced peritoneal diffusion in OC animal experimental models. Moreover, in this case, tumors displayed a decreased expression of c-Met and MMP2 expression and a diminished ERK phosphorylation [124].

Finally, exosome mimetics facilitate the transport of other active substances. Immune-derived exosome mimetics (IDEM) resulted in a productive attempt to treat ovarian cancer cells [125]. IDEM were produced from monocytic cells, and doxorubicin was employed as an associated drug. IDEM demonstrated greater encapsulation effectiveness and a higher ability to deliver doxorubicin than normal Exo. The study of doxorubicin-armed exosomes’ cytotoxic and pro-apoptotic action showed IDEM as a more effective platform than free doxorubicin and able to reduce side effects [125].

Finally, exosomes can transport substances implicated in antigen presentation, such as MHCI, HSP, and tumor-associated antigens, suggesting the possibility of an EOC immune treatment. For example, Andre et al. demonstrated that exosomes taken from malignant ascites represent a source of cancer antigens that could be transported to DCs, favoring the proliferation of cancer-specific cytotoxic T lymphocytes, provoking an anticancer response [126]. A phase I/II clinical trial evaluated the therapeutic feasibility of ascites-originated exosomes linked to TLR3 agonists for the immune treatment of EOC [127].

The fast dissemination of EOC causes several treatment challenges. Exosomes can stimulate cell angiogenesis, immunosuppression, division, and tissue invasion, crucial for EOC progression. Moreover, exosome protein signatures that are derived from EOC have been recognized. Clinically, these data may allow appropriate modifications in treatment before the onset of diffuse disease evolutions. Evaluation of the differential expression of metastatic genes derived from circulating exosomes might represent an easy screening tool for EOC detection and longitudinal monitoring of changes associated with metastatic spread. Finally, exosomes are also attractive candidates for drug delivery. At the same time, studies suggested that miRNA replacement therapy using exosomes derived from non-malignant cells may be a promising tool for EOC treatment.

#### Exosomes and Chemoresistance in Ovarian Cancer

OC has one of the highest rates of chemoresistance-related relapse: over 50% of all OCs in 5 years. Exosomes have a relevant effect in inducing this phenomenon as exo-miRNAs can control the gene activation of target cells and can favor chemoresistance in OC cells.

Li et al. demonstrated higher concentrations of miRNA-429 in multidrug-resistant SKOV3 cells and their exosomes (SKOV3-EXO) than insensitive A2780 cells and their exosomes [128]. Exo-miRNA-429 augmented chemoresistance by acting on the calcium-sensing receptor (CASR)/STAT3 pathway. Interestingly, the NF-*κ*B block decreased the production of miRNA-429 and restored the sensitivity of EOC cells. Resistant cells pretreated with an NF-κB inhibitor or miRNA-429 antagomir displayed sensitivity to drugs such as cisplatin and showed reduced cell growth [128].

Similarly, serum exosomal concentrations of miRNA-214-3p were remarkably augmented in platinum-resistant EOC cells. Transfecting the miRNA-214-3p inhibitor in EOC cells, cell growth was reduced, whereas programmed cell death was augmented. This effect appears to be due to altered mitochondrial functions caused by disproportionate ROS generation [129].

miRNA-1246 production was also remarkably significant in PTX-resistant OC exosomes than insensitive counterparts, and experimentation demonstrated that the Cav1 gene, which is the target of miRNA-1246, is implicated in exosomal transport [130]. Augmented expression of Cav1 and anti-miRNA-1246 therapy considerably sensitized OC cells to PTX. The study demonstrated that miRNA-1246 blocks Cav1 and operates via the PDGFβ receptor. Moreover, miRNA-1246 inhibitor therapy combined with chemotherapy caused a diminished cancer burden in vivo [130].

A different possibility to induce cisplatin resistance was identified by evaluating the genomic DNA methylation pattern. DNA methyltransferase 1 (DNMT1) is responsible for genome-wide methylation. DNMT1 transcripts were incredibly augmented in exosomes from ovarian cells, and in vivo employment of DNMT1-containing exosomes increased chemoresistance and xenograft progression and diminished overall survival. However, therapy with exosome inhibitor GW4869 reestablished sensitivity in resistant cells [131].

Finally, hypoxia, an essential element favoring the diffusion and aggressivity of OC, increases chemoresistance [132]. Rab proteins are a group of small GTPases that influence cell exosome production and are implicated in tumor growth and diffusion [133]. Exosomes released in the hypoxic cancer milieu may participate in these processes by transferring miRNAs and proteins between tumor cells and normal cells. A study reported that OC cells subjected to hypoxia remarkably augmented exosome discharge by increasing Rab27a, decreasing Rab7, LAMP1/2, and NEU-1. STAT3 knockdown in OC cells decreased exosome liberation by modifying the concentrations of Rab7 and Rab27a under hypoxic conditions.

Moreover, exosomes from ascites of OC patients under hypoxia transported more powerful oncogenes. Hypoxic OC cells originated exosomes efficiently modify the fallopian tube epithelial cells to be pro-oncogenic in animal models. Finally, cisplatin efflux through exosomes was significantly augmented in OC cells under a hypoxic situation. Preventing exosome liberation by amiloride or STAT3 inhibitors caused a marked increase in programmed cell death, decreased colony formation, and cisplatin-treated OC growth [134].

### 2.7. Exosomes and Pancreatic Cancer

Pancreatic cancer (PC) is digestive cancer with the worst outcome. The 5-year overall survival is less than 5% [135].

As in the case of the solid neoplasms previously described, it is also possible to transfer substances with therapeutic activity by exosomes in the case of PC. In a study, gemcitabine (GEM) was stored into autologous exosomes to generate ExoGEM to treat PC. Exosomes favored cellular absorption of GEM and determined augmented cytotoxic activity of GEM. In experimental animal models, exoGEM treatment reduced cell proliferation, with prolonged survival and insignificant injury to normal tissues. Furthermore, PC in animals treated with ExoGEM regressed without relapse [136]. Similarly, Zhou et al. armed exosomes with PTX and GEM, and these exosomes displayed exceptional cell entering capacities with significant therapeutic effectiveness [137]. A different experiment confirmed that the release of PTX-armed exosomes significantly reduced the growth of CFPAC-1 pancreatic cell lines [138].

Several other reports proved exosomes’ capacity to transport and release different molecules that target cancer cells or genes implicated in PC onset and progression. For instance, it was reported that exosomes could transport Curcumin in PC cells with in vitro cytotoxicity [139]. Furthermore, to counter gemcitabine resistance in PC, Aspe et al. employed exosomes to transport survivin T34A to PC cell lines, restoring sensitivity to gemcitabine [140].

The combined use of drugs such as GEM, all-trans retinoic acid (ATRA), and sunitinib (usually employed for PC therapy) with vaccines including DCs stocked with PC-originated exosomes proficiently blocked the diffusion of metastases and augmented animals’ survival [141].

Targeting macrophages is an encouraging method for PC treatment. Su et al. demonstrated that exosomes originating from Panc-1 cells induced a change in M1 macrophages to assume an M2 phenotype. However, this could be reversed by administering the M2 macrophages exosomes from Panc-1 cells that have been transfected with miRNA-155 and miRNA-125b2. In this case, the M2 cells reverted to an antitumor M1 phenotype [142].

A therapeutic attempt was to use a biomimetic tactic based on membrane fusion. Deng et al. generated a platform by combining CLT (Celastrol)-armed PEGylated lipids with the DC2.4 cell membrane (M-LIP-CLT) applied for the treatment of Kras-mutant PC with exceptional anti-tumor efficacy [143]. This hybrid nanoplatform combines the benefits of artificial lipids and natural cell membranes to mimic the bio-features of exosomes. Being partly PEGylated liposomes, M-LIP could include a significant quantity of CLT and have a prolonged circulation time. At the same time, being partially cell-membrane vesicles, they mimic the surface elements of exosomes for a more remarkable ability to target Kras-mutant PC cells via micropinocytosis.

Cancer-originated re-assembled exosomes (R-Exo) were employed as a drug transporter and an immunostimulatory mediator. A chlorin e6 photosensitizer was stored into cancer-originated exosomes during exosomal re-assembly. Chlorin e6-loaded R-Exo (Ce6-R-Exo) can produce ROS in cancer cells proficiently under laser irradiation. Moreover, Ce6-R-Exo augmented the production of cytokines by immune cells, which suggests that these specific exosomes can be employed for immunotherapy [144].

A different therapeutic attempt is to modify miRNAs enclosed in exosomes. Amongst these miRNAs, miRNA-124 was particularly interesting in PC patients. Some reports have demonstrated that miRNA-124 production diminishes in PC subjects, and K-Ras has been recognized as a direct target of miRNA-124. Moreover, enhancer of zeste 2 polycomb repressive complex 2 subunit is a histone methyltransferase that regulates the methylation of H3K27me3 in PC and has been reported to be a downstream gene of K-Ras. A study evaluated the anticancer effects of miRNA-124 delivered by BM-MSC-derived exosomes. To assess the effects of miRNA-124 against PC, the miRNA-124-3p mimic and pcDNA3.1-EZH2 were transferred into AsPC-1 and PANC1 cells. The miRNA-124 mimic transfection remarkably augmented the intracellular miRNA-124 concentrations, while the transfection of pcDNA3.1-EZH2 significantly augmented the generation of EZH2. Augmented levels of miRNA-124 reduced cell survival and increased programmed cell death, while the increase in EZH2 inverted this effect. Moreover, augmented levels of miRNA-124 decreased the diffusion and EMT of PC cells [145].

miRNA-34a inhibits several families of proteins, which are implicated in cell proliferation, and apoptosis. In addition to targeting Bcl-2, an apoptotic controller, miRNA-34a, reduces the production of Yin and Yang 1 (YY1) protein and Notch1/2 [146]. Exosomes loaded with miRNA-34a (exomiR-34a) were able to decrease Bcl-2 production, reduce PC cell proliferation, and cause programmed cell death in tumor cells. In vivo experiment performed in xenograft nude mice carrying Panc28 tumor cells demonstrated that exomiR-34a inhibited PC proliferation [147].

miRNA-126-3p also blocked PC proliferation and diffusion originating from BMSCs exosomes by reducing ADAM9 [148].

However, other non-coding genetic material could be a proper therapeutic target besides miRNAs. Several data have demonstrated that circRNAs were abnormally present in PC cells and contributed to the onset and diffusion of PC [149]. SCL/TAL1 interrupting locus (STIL) is the parent gene for hsa_circ_0000069, and its augmented expression was correlated to a bad outcome in PC subjects. Moreover, a decrease in hsa_circ_0000069 reduced STIL expression, diminished the diffusion and invasion of PC cells, stimulated programmed cell death, caused a cell cycle stop, and inhibited the proliferation in PA cells. Furthermore, hsa_circ_0000069 knockdown blocked the expansion of xenograft PC in vivo [150]. Pancreatic duct epithelial cells internalize SW1990 cell-originated exosomes, allowing the transport of hsa_circ_0000069. Remarkably, SW1990 cell-originated exosomes stimulated the growth of pancreatic duct epithelial cells, while exosomes with decreased hsa_circ_0000069 inhibited the growth [150].

P21-activated kinase 4 (PAK4), a component of the PAK group of serine/threonine kinases which operate as mediators of numerous small GTPases, has oncogenic activity when augmented, stimulating cell growth and diffusion. PAK4 is overexpressed in PC cells, and increased PAK4 expression is parallel with the expanded production of c-Met and the p85α subunit of PI3K. Thus, PAK4 might be a therapeutic target in PC, as demonstrated by administering Exo-mediated RNAi [151]. Intra-tumoral administration of Exo-siPAK4 decreased PC proliferation in vivo and augmented animal survival, with little toxicity [151].

Other experiments have confirmed the possibility of blocking the expression of specific genes to treat PC cells. Kamerkar et al. modified exosomes to transport siRNA or shRNA aiming KRAS in PC cells, which efficaciously blocked cancer proliferation in experimental animal models and augmented the overall survival [152], while Mendt et al. employed exosomes armed with siRNA to target KRAS G12D, which extended the survival in different PC mouse models [153].

#### Exosomes and Chemoresistance in Pancreatic Cancer

Cancer-associated fibroblasts (CAFs) regulate chemoresistance by transporting exosomal miRNAs to tumor cells. It has been demonstrated that CAFs were intrinsically resistant to gemcitabine (GEM), and the exosomes originating from CAFs participated in the onset of GEM resistance. MiRNA-106b concentration was augmented in CAFs exosomes after GEM treatment, and pretreatment miRNA-106b inhibitor favored a reduction in chemoresistance of PC cells to GEM [154].

We report in Table 1 the few ongoing clinical trials on the possible use of exosomes in the treatment of some solid neoplasms (Table 1) [155].

## 3. Exosomes and Leukemia

Leukemia-originated exosomes transport molecules that increase cellular growth and augment programmed cell death of leukemic cells.

### 3.1. Acute Myeloid Leukemia and Exosomes

Acute myeloid leukemia (AML) is a hematologic neoplasm distinguished by a high degree of alteration of differentiation and by abnormal growth of myeloid progenitor cells.

The possibility to use exosomes for leukemia treatment has been proved by several experiments [156]. Dibavar et al. treated NB4 cell line with bone marrow (BM)-hMSC-originated exosomes and arsenic trioxide, a well-known drug employed in promyelocytic leukemia. NB4 cells displayed more significant amounts of programmed cell death markers than NB4 cells exposed to exosomes or arsenic trioxide alone [157]. However, conflicting data exist in the literature. A pro-tumoral action of BM-MSC-exo has been found in a study showing that BM-MSC-exo augmented the number of leukemia stem cells (LSCs) and stimulated the discharge of leukemic cells into the peripheral blood. Probably, this effect is due to an increase in S100A4 by BM-MSC-exo into leukemic cells. S100A4 is a calcium-binding protein involved in controlling different biological functions such as cell proliferation, angiogenesis, cell diffusion, and apoptosis. The reduction in S100A4 decreased the growth and distribution of leukemia cells after BM-MSC-exo treatment [158].

Interestingly, Peng et al. detected that miRNA-34c-5p, a miRNA controlling the senescence process, was remarkably decreased in AML stem cells compared to normal hematopoietic stem cells [159]. This lower expression in LSCs was directly related to a bad response to AML treatment. Conversely, high levels of miRNA-34c-5p stimulated LSCs senescence ex vivo, inhibited leukemia onset, and augmented the suppression of LSCs in immune-deficient animals. The mechanism is related to the role of miRNA-34-5p in causing LSC aging via two different pathways: p53-p21Cip1-cyclin-dependent kinase (CDK)/cyclin and p53-independent CDK/cyclin pathways. MiRNA-34c-5p-loaded exosomes were particularly effective in favoring the aging process. Furthermore, miRNA-34c-5p augments its intracellular concentration through RAB27B, a substance that stimulates exosome detaching [160].

Thus, in-depth studies have examined the role of exosomes produced by AML blasts and how the bone marrow microenvironment changes in favor of leukemia progression and survival to identify the mechanisms of treatment failure. Findings demonstrated that malignant cells could modify the behavior of adjacent cells, and by secreting exosomes containing immune-inhibiting cytokines, AML cells render inoperative the immune system against leukemic cells, guaranteeing their survival. Thus, leukemia-derived exosomes support clonal cell growth while suppressing normal hematopoiesis; therefore, inhibiting the generation of these exosomes as well as their reprogramming seems to be an interesting approach in leukemia treatment.

Finally, exosome-based therapy has been used to stimulate an immune response against AML. Stimulated NK cells generate significant quantities of exosomes that contain important cytotoxic molecules, including IFN-*γ*, lymphocyte function-associated antigen (LFA-1), DNAX accessory molecule-1 (DNAM1), and programmed cell death protein (PD-1) [160]. Vaccines with DCs electroporated with Wilms Tumor 1 mRNA have displayed high effectiveness in reducing relapses in AML subjects [161]. In this context, DCs pulsed with exosomes induced an effective cytotoxic activity and antileukemic immune response in mice experimental models. In the specific case of AML, DCs pulsed with exosomes isolated from leukemia cell cultures caused leukemic regression and prolonged survival of vaccinated animals [162]. Unexpectedly, in vitro incubation of exosomes with patients’ DCs reduced K562 cell death, whereas the destruction of leukemic cells was significantly augmented when immature DCs were pulsed with exosomes originating from K562 cultures [163]. Therefore, according to this study, the employment of vaccines utilizing patients’ exosomes would not lead to the appearance of a beneficial effect. In contrast, exosomes from cultured cells may induce a DCs cytotoxic phenotype.

#### Exosomes and AML Chemoresistance

The effect of exosomes inducing resistance to various chemotherapeutic drugs such as doxorubicin and etoposide is decisive. For instance, U937 cells augment their resistance against the PEGylated liposomal doxorubicin (PLD) via the exosome-related discharge of the PLD. Blocking exosome delivery could stop PLD emission and raise the susceptibility of U937 cells to the toxic actions of PLD. U937 cells were administered with PLD with or without the dispensation of the exosome discharge blocking agent, GW4869. Inhibitor administration augmented cell death. Remarkably, the administration of GW4896 and 0.5 μM PLD induced the equivalent cytotoxic action as that of the 1 μM PLD [164].

In a further study, it has been reported that exosomes isolated from AMLs stimulate IL-8 production in HS-5 BMSCs, which causes etoposide resistance in AML cells. Blocking exosomes or IL-8 can revert the chemoresistance, with augmented etoposide-induced apoptosis of AML cells [165].

### 3.2. Exosomes and Acute Lymphoblastic Leukemia

Numerous evidence has proved that exosome-originated miRNA-181a affects central nervous system alteration in pediatric acute lymphoblastic leukemia (PALL), and the use of an miRNA-181a inhibitor may produce beneficial effects in PALL [166]. Blocking exo-miR-181a caused the decrease in genes able to augment cell growth, such as BCL2, PCNA, MCL-1, and KI-67. Moreover, inhibiting pro-apoptotic genes such as BAX or BAD can reduce exosome-caused cell growth [167].

Anti-leukemic immune responses can also be obtained for ALL by administration of leukemia-originated exosomes. The shRNA inhibition of TGF-β1 in ALL cell lines efficaciously decreased the TGF-β1 concentration in exosomes originated from leukemic cells (LEX). When LEXTGF-β1si were absorbed by DCs, they were able to stimulate DC activities by augmenting the presence of MHC class II molecules. Moreover, they caused the production of TNF alpha and IL-12p70 and augmented CD4+ T-cell growth and Th1 cytokine production. Finally, they induced a powerful T lymphocyte and NK cell cytotoxic response. In experimental animal models, administration of LEXTGF-β1si provoked an inhibition of leukemia cell proliferation and prolonged survival [168].

Exosome-based immunotherapies are safe and effective. For instance, CAR-T cell treatment is burdened with numerous side effects, such as cytokine storms. Haque et al. generated exosomes presenting CD19 CAR to treat CD19-positive B-cell neoplasms. This approach decreased the risk of excessive cytokine discharge and was associated with cellular toxicity and augmented programmed cell death genes in CD19 leukemia B-cells without causing apoptosis in CD19-negative cells [169].

#### Exosomes and Acute Lymphoblastic Leukemia Chemoresistance

Relapsed leukemia is generally due to the therapy failure due to chemoresistance. Vincristine and prednisone are drugs employed for the ALL therapy, as they cause programmed cell death in first-line leukemia. However, these drugs do not provoke apoptosis in relapsed leukemia. This is probably due to the inability to determine suppression of cellular and exosomal miR-181a expression. These findings propose a possible effect of an inhibitor on exosome-derived miRNA-181a in the treatment of relapsed ALL [170].

### 3.3. Exosomes and Chronic Myeloid Leukemia

Chronic myeloid leukemia (CML) is a malignancy distinguished by a reciprocal translocation between the long arms of chromosomes 9 (ch9) and 22 (ch22), which determines the production of the Philadelphia chromosome. Even though the insertion of tyrosine kinase inhibitors (TKIs) transformed treatment and prognosis in CML patients, the use of new therapeutic approaches could further improve the natural history of this disease.

In a study, a venetoclax-armed immunoliposome (IL-VX) was designed and showed a more remarkable ability to induce CML programmed cell death than free venetoclax [171]. Another study reported the effects of exosomes armed with imatinib and IL-3 on CML blasts being interleukin 3 receptor augmented on CML blasts. IL3-exosomes armed with imatinib target CML blasts and transported imatinib and BCR-ABL1-silencing RNA, with a consequent reduction in leukemia cell proliferation in vitro and in vivo and imatinib-resistant cells [172].

Several other substances might be used to arm exosomes against CML cells. Curcumin has been reported to display antitumor activities in many tumors [173]. The addition of Curcumin to CML cells provoked a dose-dependent augmentation of phosphatase and tensin homolog (PTEN), an oncosuppressor gene, target of miRNA-21. Curcumin administration also reduced Akt phosphorylation and VEGF production, affecting the survival of CML cells. A report evaluated the miRNA-21 amount in K562 and LAMA84 cells and exosomes after the addition of curcumin and its effect on CML cells. Authors demonstrated that the addition of curcumin to CML cells provoked a reduction in Bcr-Abl expression via the cellular increase in miRNA-196b. The action of curcumin was also evaluated on a CML xenograft in SCID mice, and curcumin-administered animals presented a lower tumor burden compared to controls. Moreover, exosomes discharged in the plasma of the curcumin-administered animals presented augmented amounts of miRNA-21. These findings indicated that a storing of miRNA-21 in exosomes might participate in the antitumoral action of curcumin in CML [174].

It was also reported that curcumin exosomes reduce Ras homolog gene family member B (RhoB) production and negatively control endothelial cells diffusion. Adding CML exosomes to HUVECs provokes an augment in VCAM1 and IL-8 concentrations, but curcumin-loaded exosomes inverted these results, reducing their angiogenic effects. This antiangiogenic property was verified with in vitro and in vivo studies. Quantitative analyses of peptides of curcumin exosomes demonstrated that curcumin addition modifies their molecular characteristics. Curcumin stimulates the discharge of exosomes depleted of pro-angiogenic factors and enriched in proteins with antiangiogenic functions. Among these factors, Myristoylated alanine-rich C-kinase substrate (MARCKS) seems to play an essential role since it was a target of the miRNA-21 mentioned above. Thus, curcumin reduces the exosome’s capacity to stimulate the angiogenic phenotype and regulate the endothelial barrier structure [175].

Other pathways could also be involved in exosome-intermediated treatment, such as the transforming growth factor (TGF)-*β*1, enhanced in LAMA84-originated exosomes. Raimondo et al. assessed the autocrine effects of exosomal-TGFbeta1 on leukemic cells employing a TGF-*β*1 receptor inhibitor or a neutralizing antibody. They demonstrated that the exosome augmented cell growth, and the generation of an antiapoptotic phenotype can be avoided by inhibiting the TGF-*β*1 system [176].

Finally, Zhang et al. reported that BM MSC-Exo could block the growth of CML cells in vitro through miRNA-15a and induce apoptosis. These results were not achieved in BALB/c nu/nu mice. BMMSC-Exo augmented CML occurrence and stimulated tumor diffusion in vivo. It was reported that the production of the antiapoptotic protein Bcl-2 increased, while the caspase3 generation reduced [177]. Further studies are needed to understand the reason for these discrepancies

#### Exosomes and CML Chemoresistance

Imatinib resistance is the greatest obstacle for the therapy of CML, and recently there was the observation that exosomes can affect drug resistance. It was demonstrated that hUC-MSC-Exo alone had no action on cell survival or programmed cell death of K562 951 cells. However, hUC-MSC-Exo promoted IM-induced cell viability inhibition and apoptosis. Moreover, hUC-MSC-Exo enhanced the increased Bax expression and the decreased Bcl-2 expression that IM induced. Interestingly, the antitumoral actions of hUC-MSC-Exo on K562 cells could be blocked by treatment with caspase inhibitor Z-VAD-FMK [178].

In a different study, authors reported that miRNA-328 remarkably was reduced during the onset of imatinib resistance [179]. MiRNA-328 supplementation reverted the resistance to imatinib by reducing ABCG2 expression, while miRNA-328 knockdown caused imatinib resistance in K562 cells. Furthermore, the transfer and discharge of alkalized exosomes augmented miRNA-328 production and sensitized the CML cells to imatinib.

## 4. Exosomes and Lymphomas

Lymphomas are lymphoid malignancies that originate from lymph glands or extra-nodal lymphoid tissue [180].

Several substances can be inserted into exosomes for therapeutic purposes against lymphoproliferative diseases. Curcumin stored in lymphoma cells-originated exosomes target CD11b^+^/Gr–1^+^ cells, induces an increase in programmed cell death, and has anti-inflammatory activity [181].

Omacetaxine, previously identified as homoharringtonine (HHT), is a natural alkaloid originating from *Cephalotoxus fortunei* with an anticancer activity that operates synergistically with different drugs in non-Hodgkin lymphoma (NHL) via NOXA and MCL-1-dependent systems [182]. HHT interferes with angiogenesis and tumor growth in lymphomas [183]. A study stated that HHT and curcumin remarkably blocked the development and diffusion in U937 and Raji cells and reduced VEGF concentration in the same cells. In the meantime, combined use of the two substances reduced VEGFA concentrations in exosomes that originated from Raji cells. Treatment with exosomes with a low amount of VEGF to HUVECs inhibited growth and tube formation of HUVECs, and diminished MMP2, MMP9, p-Akt, and angiogenin-1. This suggested that combined administration of HHT and curcumin could block lymphoma cell proliferation and angiogenesis through repression of the VEGF/Akt system [184].

Koch et al. reported that treating diffuse large B-cell lymphoma (DLBCL) cell lines with a non-steroidal anti-inflammatory drug such as indomethacin leads to reduced exosomes discharge and inhibited lymphoma development. Moreover, they proved that a reduced exosome discharge causes an augmented efficacy of drugs such as anthracyclines, both in vitro and in vivo [185]. A different approach involves the decrease in exosomes’ absorption by inhibiting elements such as heparan sulfate proteoglycans (HSPGs) on target cells [186]. HSPGs have been proposed to operate as a receptor for exosome absorption. It was proved that pretreatment of exosomes with low molecular weight heparin drastically reduced the absorption of chronic lymphocyte leukemia-derived EV by target cells augmenting cell sensitivity [187,188].

A further attempt was to cause programmed cell death of lymphoma cells employing TRAIL-mediated apoptosis. Numerous molecules affecting this system have been identified, comprising TRAIL receptor agonists and recombinant soluble TRAIL. However, no substantial antitumor effects could be demonstrated in tumor patients. A novel possibility to transfer and discharge TRAIL could be to insert it within exosomes produced by TRAIL-expressing cells. Rivoltini et al. reported the capacity of TRAIL-loaded exosomes to move molecules able to reduce cell proliferation and stimulate programmed cell death in lymphoma cells [189]. Intratumor administration of TRAIL+ exosomes provoked a reduced increase in SUDHL4. Moreover, TRAIL+ exosomes cluster in tumor sites and in the lungs, spleen, and liver, generating a substantial decrease in lymphoma proliferation in SUDHL4-bearing mice. Interestingly, programmed cell death related to TRAIL exosomes was inhibited by adding TRAIL Ab [189].

The inhibition of numerous genetic pathways induced by modifying the activity of the exosomes could constitute a practical therapeutic possibility. Lunavat et al. generated exosome-like nanoparticles containing siRNA and demonstrated that inhibiting c-Myc effectively stimulated poly (ADP-ribose)polymerase-dependent apoptotic systems in l820 lymphoma cells [190]. Moreover, the shRNA approach has been employed to inhibit TGF-beta1 in lymphoma cells, augmenting the discharge of TGF-beta1-depleted exosomes. By eliminating this antitumor–immune surveillance inhibitor, it was possible to stimulate an increase in the immune response against leukemic cells [165]. Modified exosomes were also employed to transport and deliver tumor antigens to elicit immune responses. In a study, stromal cells were transfected with the Epstein–Barr virus protein gp350. These cells were able to discharge gp350C exosomes interacting with CD21 on B cells. The absorption of Gp350C exosomes in chronic lymphocytic leukemia cells caused a strong immunogenic effect, triggering the stimulation of tumor-associated and EBV-specific T-cells [191].

Synthetic lipid vesicles loaded with active Apo2 ligand/TRAIL were evaluated on lymphoma cell lines with relevant pro-apoptotic action of cancer cells without effects on normal cells both in vitro and in vivo, with little toxicity in vivo [192].

However, the possibility to manipulate exosomes to restore anti-lymphoma immunity was also confirmed in numerous other models of lymphoma. An exosome-DC vaccine was constructed to reestablish immune surveillance in lymphoma. Unlike what was described when DLBCL-originated exosomes were incubated with T-cells and caused their inhibition by producing different immunosuppressive substances [193], DLBCL-derived exosomes transporting lymphoma-specific antigens pulsed into DCs reestablished the antigen-presenting ability of DCs, provoking the onset of powerful CTL-dependent lysis of lymphoma cells [194].

The capacity of exosomes to bind to particular receptors on both lymphoma cells and microenvironment cells via membrane molecules renders them an attractive instrument to transport exogenous cytotoxic and inhibitory molecules for therapeutic intents. Engineered exosomes are potent tools to be utilized against B-cell tumors. As stated above, it is clear that one of the several vantages is the possibility to join this approach with other strategies, such as traditional chemotherapy and those intending at rebooting the host immune system. These approaches have the potential to reduce tumor growth and diffusion, thus decreasing any off-target side effects.

## 5. Exosomes and Multiple Myeloma

Multiple myeloma (MM) is classified by the clonal growth of neoplastic plasma cells and the production of monoclonal immunoglobulin in the BM. MM is still judged as an incurable malignancy [195,196].

MM is a disease with a highly destructive action on the BM. The delivery and discharge of exosomes MM plasma cells considerably modify the BM milieu, stimulating plasma cell proliferation and the onset of lytic lesions. This changed microenvironment establishes a particular place, a niche, which is superlatively appropriate for maintaining its growing elements and establishing a reduced immune response and chemoresistance [197].

Several reports have evaluated the possibility to modify exosome production to target MM plasma cells. In animal MM experimental models, sphingomyelinase inhibitor GW4869 inhibited exosome discharge, avoiding the onset of exosome-mediated lytic lesions and augmenting cortical bone volume. GW4869 also powerfully operated synergistically with bortezomib to provoke anti-myeloma effects, proposing that modifying exosome transport and delivery can influence MM growth and viability. However, ceramide C6 (C6-cer), an exogenous ceramide, augmented MM exosome production but blocked cell growth and caused programmed cell death [198].

Different molecules could help modify the generation and activities of exosomes in MM. For example, a noteworthy molecule is a protein called heparanase, supplied through the production of exosomes. Heparanase, produced both by MM or host cells, reaches the BM milieu and controls the activities of adjacent cells by stimulating the production of exosomes. It modifies exosome contents inducing the production of cytokines correlated to the onset of MM or the generation of blood vessels, such as VEGF, syndecan-1, and hepatocyte growth factor [199,200]. Reports have demonstrated that heparanase production in MM subjects is more outstanding, suggesting that this molecule might be a possible therapeutic target. A study by Ritchie et al. reported that a heparanase inhibitor named SST0001 reduced MM cell proliferation [201]. Another study proved that H1023, a monoclonal antibody, could block heparanase enzymatic effects [202].

Several agents can inhibit heparinase, such as modified heparin or heparin mimics, that are presently valued in clinical trials. Purushothaman et al. demonstrated that heparan sulfate exerts a relevant effect on exosome-cell communication, encapsulating fibronectin on exosomes and operating as a receptor for fibronectin. This effect can stimulate pERK and p38 pathways or MMP-9 and Dickkopf-1 involved in MM advancement [203]. The most relevant aspect of the experiment is discovering that inhibiting heparan sulfate by using monoclonal antibody for Hep-II heparin-binding domain of fibronectin or removing it with bacterial heparitinase prevents the effects of exosomes on MM cells [203]. Similarly, the heparin originated Roneparstat blocked communications between exosomes and MM cells in phase 1 clinical trial (NCT01764880) [204].

The effects of other inhibitors were also evaluated. However, although a decrease in exosomes absorption was demonstrated in BMSCs after administration of molecules such as inhibitors of membrane fusion, endocytosis, or macropinocytosis, these compounds negatively modified the survival of BMSCs. Dynasore and heparin did not alter BMSC survival, while substances such as amiloride and omeprazole remarkably reduced BMSC survival. Moreover, they seemed to act via the inhibition of STAT1, STAT3, and ERK1/2 phosphorylation in the presence of RPMI8226 or H929 exosomes [205].

The study of exosome activity after treatment could open new, unexpected fields of knowledge in MM [206]. Many drugs used in the treatment of MM, or other neoplasms, could use exosomes as valuable vehicles for modifying the cellular activity of neoplastic cells and the medullary microenvironment. In an elegant study, Malavasi et al. analyzed the membrane changes of MM cells induced by specific antibodies targeting CD38 [204]. MM cells (BF01) were cultured in vitro with antibodies, and exosomes originated from antibody-exposed cells increased CD73 and CD39 expression, programmed death-ligand 1, and induced relevant changes of miRNAs production. Moreover, after exosome absorption, NK cells presented a decrease in genes implicated in the cell cycle and an augmented expression of genes correlated to the stimulation of immune response [207].

Different mechanisms, such as an extra activity of immune effectors, determined by a modification of the movement of the exosomes, justify the therapeutic action of other substances. A study suggested that small dosages of doxorubicin and melphalan cause cell senescence, augmenting IL15/IL15RA complex production in MM cells and their exosomes, stimulating NK cell function, and growth [208]. Thus, chemotherapy might operate by releasing exosomes to induce an anti-myeloma immune response.

Finally, MM-originated exosomes as a source of MM antigens for vaccines have also been studied. Xie et al. demonstrated that HSP70-modified MM cell-originated exosomes induced DCs development and triggered effectual CD4^+^/Th1, CD8^+^/CTL, and NK-mediated anti-MM immune response. Moreover, membrane-bound HSP70 operated both as a damage signal and an antigenic peptide chaperone that stimulated DCs, representing an essential adjuvant for exosome-based antitumor vaccine [209].

A modulation of exosome activity could also be useful in preventing or treating numerous complications of MM. More than 50% of MM subjects present a heart injury such as heart insufficiency or cardiomyopathy provoked by amyloidosis or anemia. In addition, some treatments used for MM can affect cardiac function through specific circRNAs [210]. For instance, circ-G042080 was augmented in the serum exosomes of MM subjects and might provoke myocardial damage via the activation of the miRNA/TLR4 axis. In vitro study reported that the circ-G042080/hsa-miR-4268/TLR4 axis might operate in H9C2 cells cultured with exosomes and provoke anomalous autophagy [211]. Interfering with this system could reduce heart damage in MM patients.

### Exosomes and Chemoresistance in Multiple Myeloma

Exosomes could modify the resistance to MM treatment. Fact et al. reported an alteration in the sphingolipid metabolism, increasing ceramides and acid sphingomyelinase (ASM) and decreasing sphingomyelin [212]. Moreover, a further augment in ASM in MM cells and in their exosomes after treatment with melphalan or bortezomib was reported. Exosomes with a raised concentration of ASM can transmit the chemo-resistant phenotype to chemo-sensitive cells. Furthermore, amitriptyline can extend drug sensitivity in MM cells by inhibiting ASM.

As reported above, heparanase (HPSE) is augmented in MM cells and correlates to MM cell proliferation and bortezomib (BTZ) resistance. Rodrigues-Junior et al. assessed miRNAs and HPSE presence in MM lines (U266 and RPMI-8226) [213], and synthetic miRNA mimics were inserted in MM cells to evaluate the miRNA effect in HPSE expression. Exosomes derived from HEK293T cells were modified with miRNAs to assess their impact on BTZ. The results showed a relevant correlation between BTZ sensitivity, HPSE, and miRNA-1252-5p generation. Moreover, increased production of miRNA-1252-5p remarkably diminished HPSE generation and HPSE activity in MM cells. The greater concentration of miRNA-1252-5p was related to a decrease in cell survival and a reduced chemoresistance to BTZ. Finally, exosomes transporting miRNA-1252-5p increased MM cells’ sensitivity to BTZ administration.

In conclusion, exosomes have been involved in MM-correlated processes comprising angiogenesis, osteolysis, immune suppression, and drug resistance. Pursuing exosome production and secretion could, therefore potentially inhibit these different processes.

## 6. Conclusions

In recent years, a wide variety of drug transporting platforms, such as nanoparticles, have been implemented. Still, they present several inconveniencies, such as their xenobiotic origin, which can provoke unwanted immune reactions and unexpected toxicities [214]. On the contrary, exosomes have modest toxicity and mutagenicity and a high capacity to carry and discharge different compounds with ample distribution in the body fluids and across the blood–brain barrier. Moreover, they present other receptors and ligands which favor targeting specific cells [7] (Table S3). Furthermore, it is easy to insert genetic material into exosomes [215]. Finally, in vivo exosomes can be administered intravenously, intramuscularly, or intraperitoneally.

Exosomes have several features that make them appealing for tumor treatment. For instance, they can elude blood clearance by the immune system [216], while the nanoscopic dimensions allow their infiltration into cancer mass and metastatic sites. Lastly, the specific organotropism of exosomes to cancer sites renders them perfect elements for the institution of productive antitumor treatments with a low incidence of side effects [217].

In the near future, a fruitful field of study could be constituted by the analysis of the role played by exosomes in long-distance cell–cell communication, maybe from one organ to another. Tunneling nanotubes (TNTs) are membrane-enclosed tubular connections between cells that carry a large number of cellular cargoes, such as exosomes, non-coding RNAs, and mitochondria, and they have been recognized as the principal participants in tumoral cell communication. TNTs have been reported in several cancers in in vitro, ex vivo, and in vivo models promoting the onset and progression of tumors. Cancer cells employ TNT-like channels to transport information between themselves or with the tumoral milieu. As a result, tumor cells accomplish novel capabilities, such as the augmented capacity of metastasis, angiogenic capacity, and chemoresistance, elevating tumor severity [218].

However, despite all these favorable characteristics, some inadequacies slow their clinical employment as a delivery system. The main problem is the difficulty of a large-scale preparation: the generation of subject-originated exosomes is expensive, time consuming, and challenging. Moreover, the reproducibility represents a different difficulty, as experimental data suggest that three diverse formulations of exosomes from MSC only shared 20% of their proteome.

An encouraging option for exosome-founded cancer treatments is manufacturing exosome mimetics, which could allow the synthesis of exosomes appropriate for clinical employment.

A different source of exosomes could be plants. Plant exosomes show similar characteristics to those of mammalian exosomes. Plant exosomes have been defined as “exosome-like” as their appearance and size are comparable to mammalians. Numerous experiments have demonstrated that edible plant-originated exosomes can aggregate in mammalian cells, while plant microRNAs might operate as controllers of biological functions in mammals. In particular, plant-derived exosomes have antitumor activity, as in the case of Moringa oleifera seed exosomes [219] or Citrus Limon L. enclosed nanoparticles (whose features make them exosome-like nanovesicles [220].

The ability of exosomes to augment the radiation resistance of cancer cells opens exciting perspectives for their use in tumor treatment [221,222]. In contrast, exosomes might affect the mechanism of action of a new class of immunotherapy such as the oncolytic virus [223,224]. Exosomes might also have relevant activity in the abscopal effect, a condition in which immune stimulation is the main factor. The research evaluated the role of exosomes in the abscopal effect of a telomerase-specific oncolytic adenovirus, Telomelysin (OBP-301). Exosomes separated from HCT116 human colon carcinoma cells treated with OBP-301 demonstrated an increased programmed cell death and autophagy comparable to OBP-301. In different cancer experimental models, an intra-tumoral dispensation of OBP-301 provoked decisive anticancer actions on tumors that were not directly treated with OBP-301, implicating straight intervention by cancer-originated exosomes enclosing OBP-301 [225].

In conclusion, exosomes are essential mediators and regulators of cellular communication. However, a complete full comprehension of the interchange between the cancer milieu and the exosome is necessary to apply them in clinical practice successfully. Identifying the exosomes’ contents is not enough to plan an exosome-associated cancer treatment but comprehending the communication patterns and forms of communication might be the key.

## Figures and Tables

**Table 1:** Ongoing clinical trials on the use of exosomes in the treatment of some solid neoplasms.

Condition	Study Title	Interventions	ID
Advanced Breast Cancer	Omic technologies to track resistance to palbociclib in metastatic breast cancer	Procedure: specimen sample collection	NCT04653740
Non-small cell lung cancer	Trial of vaccination with tumor antigen-loaded dendritic cell-derived exosomes	Biological: Dex2	NCT01159288
Colon cancer	A study investigating the ability of plant exosomes to deliver curcumin to normal and colon cancer tissue	Dietary Supplement: Curcumin conjugated with plant exosomes	NCT01294072
Pancreas cancer	iExosomes in treating participants with metastatic pancreas cancer with KRASG12d mutation	Drug: mesenchymal stromal cells-derived exosomes with KRASG12D siRNA	NCT03608631

## Data Availability

Not applicable.

## Supplementary Materials

The following are available online at https://www.mdpi.com/article/10.3390/cells11071128/s1, Table S1: The main molecules involved in promoting cancer development in different tumours and in the oncosuppressor process in different tumours, Table S2: The main molecules involved in the drug resistance for each cell exosome, Table S3, The molecules with a therapeutic potential for each cell.
