# Supplementary material for: Exosome-Mediated Therapeutic Strategies for Management of Solid and Hematological Malignancies
Source: Cells. 2022 Mar 27;11(7):1128. doi: 10.3390/cells11071128 (PMC8997895; doi:10.3390/cells11071128)
Supplement: Supplementary file 1 [file cells-11-01128-s001.zip › cells-1614916-supplementary.pdf]

Supplementary Table S1. The main molecules involved in promoting cancer development in different tumours and in the oncosuppressor process in different tumours. BC breast cancer exosomes, LC lung cancer exosomes, CC colorectal cancer exosomes, HCC hepatocellular cell exosomes, EOC epithelial ovarian cell exosomes, PC pancreatic cell exosomes, LSC leukemia stem cell exosomes, ALL acute lymphoblastic leukemia cell exosomes.

Pro-oncogenic non-coding RNAs	Pro-oncogenic proteins	Oncosuppressor non-coding RNAs	Oncosuppressor proteins
**BC**			
miR-106a-5p	-	LNA anti-miR-144-3p, miR-126, miR-130, miR-100	-
**LC**			
let-7a-5p	hsp-90	miR-497, miR-26b	-
**CC**			
miR-183-5p, miR-2277-3p	-	miR-26b-3p	-
**HCC**			
miR-21	-	Sphk2 siRNA, miR-26a, miR-335-5p, miR-142, miR-223	-
**EOC**			
miR-429, miR-214-3p	-	miR-7, miR-101, miR199a-3p	-
**PC**			
miR-106b	-	mIR-155, miR125b2, miR-124, miR-34a, miR-126-3p	-
**LSCs**			
miR-181a	S100A4	miR-34c-5p	-
**ALL**			
-	-	shRNA	-
**CML**			
-	-	miR-196b,miR-21, miR-15a	-

Supplementary Table S2. The main molecules involved in the drug resistance for each cell exosome. Particularly, we highlighted their role in inducing or reducing chemoresistance. BC breast cancer exosomes, LC lung cancer exosomes, CC colorectal cancer exosomes, HCC hepatocellular cell exosomes, EOC epithelial ovarian cell exosomes, PC pancreatic cell exosomes, AML acute myeloid leukemia cell exosomes, ALL acute lymphoblastic leukemia cell exosomes, CML chronic myeloid leukemia cell exosomes, MM multiple myeloma exosomes.

Exosomes and drug-resistance	
**BC**	CXCR4+TRAIL synergic with carboplatin<br>miRNA-567 synergic with trastuzumab<br>mIR-770 synergic with doxorubicin
**LC**	miRNA-34a and K-ras siRNA +++ response<br>miRNA373, miRNA146a-5p, miRNA512<br>- - - resistance to cisplatin<br>miR-130a +++ chemoresistance<br>lncRNA H19 +++ resistance
**CC**	-
**HCC**	lncRNA - - - sorafenib sensitivity<br>miR-199a +++ doxorubicin sensitivity
**EOC**	miRNA-429<br>miR-214-3p<br>miR-1246<br>+++ chemoresistance
**PC**	miRNA-106b +++ chemoresistance
**LSCs**	-
**AML**	exosome discharge blocking agent W4869
**ALL**	miR-181a +++ resistance
**CML**	hUC-MSC-Exo synergic with imatinib<br>miRNA-328 synergic with imatinib
**MM**	heparanase (HPSE) +++ bortezomib resistance

Supplementary Table S3. The molecules with a therapeutic potential for each cell exosome. Most of them may serve for antineoplastic drug development or as add-on treatment for already existing therapies. BC breast cancer exosomes, LC lung cancer exosomes, CC colorectal cancer exosomes, HCC hepatocellular cell exosomes, EOC epithelial ovarian cell exosomes, PC pancreatic cell exosomes, AML acute myeloid leukemia cell exosomes, ALL acute lymphoblastic leukemia cell exosomes, MM multiple myeloma exosomes.

Exosomes and therapy	
**BC**	doxorubicin<br>taxol<br>trastuzumab<br>GDEPT of 9-p amino-6-chloro-5H-benzo[a]phenoxazine-5-one
**LC**	PTX<br>withaferin A<br>celastrol<br>MRX34<br>curcumin
**CC**	sevoflurane blocked CC<br>by miR-34a-5p
**HCC**	AFP DEXs powerful<br>antigenic response
**EOC**	doxorubicin exosomes less collateral effect<br>antagomiR-429<br>antagomiR-214-3p<br>- - - resistance to cisplatin<br>antagomiR-1246 - - - resistance to PTX
**PC**	siRNA target KRAS G12D<br>antagomiR-106b
**LSCs**	-
**AML**	exosomes isolated from leukemia cell cultures
**ALL**	antagomirR-181a
**CML**	-
**MM**	miRNA-1252-5p - - - HPSE
